# Association Between DCE-MRI Perfusion Histogram Parameters and EGFR and VEGF Expressions in Different Lauren Classifications of Advanced Gastric Cancer

**DOI:** 10.3389/pore.2021.1610001

**Published:** 2022-01-06

**Authors:** Zhiheng Li, Zhenhua Zhao, Chuchu Wang, Dandan Wang, Haijia Mao, Fang Liu, Ye Yang, Feng Tao, Zengxin Lu

**Affiliations:** ^1^ Shaoxing University School of Medicine, Shaoxing, China; ^2^ Department of Radiology, Shaoxing People’s Hospital, Shaoxing Hospital, Zhejiang University School of Medicine, Shaoxing, China; ^3^ Department of Pathology, Shaoxing People’s Hospital, Shaoxing Hospital, Zhejiang University School of Medicine, Shaoxing, China; ^4^ Department of Gastrointestinal Surgery, Shaoxing People’s Hospital, Shaoxing Hospital, Zhejiang University School of Medicine, Shaoxing, China; ^5^ The First Affiliated Hospital of Shaoxing University, Shaoxing, China

**Keywords:** EGFR, VEGF, advanced gastric cancer, DCE-MRI, histogram parameters

## Abstract

**Objective:** To investigate the correlations between dynamic contrast-enhanced magnetic resonance imaging (DCE-MRI) perfusion histogram parameters and vascular endothelial growth factor (VEGF) and epidermal growth factor receptor (EGFR) expressions in advanced gastric cancer (AGC).

**Methods:** This retrospective study included 80 pathologically confirmed patients with AGC who underwent DCE-MRI before surgery from February 2017 to May 2021. The DCE-MRI perfusion histogram parameters were calculated by Omni Kinetics software in four quantitative parameter maps. Immunohistochemical methods were used to detect VEGF and EGFR expressions and calculate the immunohistochemical score.

**Results:** VEGF expression was relatively lower in patients with intestinal-type AGC than those with diffuse-type AGC (*p* < 0.05). For VEGF, Receiver operating characteristics (ROC) curve analysis revealed that Quantile 90 of Ktrans, Meanvalue of Kep and Quantile 50 of Ve provided the perfect combination of sensitivity, specificity, positive predictive value (PPV) and negative predictive value (NPV) for distinguishing high and low VEGF expression, For EGFR, Skewness of Ktrans, Energy of Kep and Entropy of Vp provided the perfect combination of sensitivity, specificity, PPV and NPV for distinguishing high and low EGFR expression. Ktrans (Quantile 90, Entropy) showed the strongest correlation with VEGF and EGFR in patients with intestinal-type AGC (r = 0.854 and r = 0.627, respectively); Ktrans (Mean value, Entropy) had the strongest correlation with VEGF and EGFR in patients with diffuse-type AGC (r = 0.635 and 0.656, respectively).

**Conclusion:** DCE-MRI perfusion histogram parameters can serve as imaging biomarkers to reflect VEGF and EGFR expressions and estimate their difference in different Lauren classifications of AGC.

## Introduction

Gastric cancer (GC) is the fifth most common cancer worldwide and the leading cause of cancer-related mortality [1]. Because of the lack of specific signs of early GC, many people are usually diagnosed at an advanced stage [2]. Currently, the treatment methods of advanced gastric cancer (AGC) mainly include preoperative chemoradiotherapy, immunotherapy, and molecular-targeted therapy, with molecular-targeted therapy emerging as an effective method to improve prognosis. Moreover, targets for targeted therapy of AGC mainly include vascular endothelial growth factor (VEGF) and epidermal growth factor receptor (EGFR) [3]. VEGF and EGFR are multifunctional cell regulatory factors that promote the formation and growth of tumor vessels, which play an important role in tumor growth, invasion, and metastasis [4,5]. Additionally, the Lauren classification has shown many differences in etiology, epidemiology, and pathology [6]. Accurate knowledge of VEGF and EGFR expressions and their differences in different Lauren classifications before operation might assist in selecting the appropriate treatment methods for patients with AGC. However, VEGF and EGFR expressions are mainly detected by immunohistochemistry (IHC), which is influenced by sampling and may not be adequately comprehensive. Therefore, there is an urgent need to develop a noninvasive and reliable method for assessing VEGF and EGFR expressions in patients with AGC.

As an emerging functional imaging technique, dynamic contrast-enhanced magnetic resonance imaging (DCE-MRI) can provide pharmacokinetic models that enable the quantification of contrast agent exchange between the intravascular and interstitial spaces and the assessment of the functional features of a target tissue [7]. Different quantitative parameters such as volume transfer constant (Ktrans), reverse reflux rate constant (Kep), extracellular extravascular volume fraction (Ve), and plasma volume fraction (Vp) can be obtained in DCE-MRI [8]. At present, DCE-MRI has proven to be reliable in predicting extramural venous invasion and patient prognosis and monitoring antiangiogenic therapies [9,10].

Histogram analysis not only calculates the average value of histogram parameters for the whole tumor but also publishes each voxel of the region of interest (ROI) to the histogram [11]. Accumulating evidence has indicated that it can appropriately quantify the heterogeneity in a tumor [12]. Thus, histogram analysis might be a noninvasive imaging method for predicting tumor biology. This study aimed to evaluate the association between DCE-MRI perfusion histogram parameters and VEGF and EGFR expressions in different Lauren classifications of AGC.

## Materials and Methods

The Institutional Review Board of Shaoxing People’s Hospital approved this retrospective study and waived the requirement for written informed consent to review the medical records and images of patients, because of the retrospective nature of this study.

### Study Participants

The imaging data of 80 patients with AGC admitted to Shaoxing People’s Hospital between February 2017 and May 2021 were enrolled in this study and analyzed retrospectively. The inclusion criteria were as follows: (1) GC confirmed by gastroscopic biopsy or postoperative pathology; (2) no absolute contraindication of magnetic resonance imaging (MRI); and (3) no anti-tumor treatment before DCE-MRI examination. In contrast, the exclusion criteria were as follows: (1) severe motion artifact contaminations in the DCE-MRI results; (2) a maximum tumor diameter of <1 cm; and (3) surgical or puncture taboos, such as coagulation dysfunction.

### Dynamic Contrast-Enhanced Magnetic Resonance Imaging Protocol

MRI studies were conducted using a 3.0T MRI scanner (Verio, Siemens, Germany) with a standard, 12-channel phased-array body coil. The preparation before scanning was as follows: (1) all patients were required to fast for 6–8 h before DCE-MRI to empty the gastrointestinal tract; (2) patients had to drink 800–1,000 ml of water to distend the stomach before MRI; and (3) anisodamine (Minsheng, Hangzhou, China) was intramuscularly administered to prevent gastrointestinal motility.

During the examination, the patient was in the supine position, where the scanning range included the whole stomach. All patients had to undergo a routine plain scan [T1-weighted image (T1WI), T2WI], followed by the DCE-MRI scan. DCE-MRI adopts free-breathing and is performed using a three-dimensional, radial volumetric interpolated, breath-hold examination technique. Multi-angle cross-sectional T1WI in the axial plane scan was initially performed with the following parameters: repetition time/echo time: 3.25 ms/1.17 ms, field of view: 350 × 284 mm, matrix: 288 × 164, layer thickness: 5 mm, scan at different flip angles (5°, 10°, and 15°) for a period of 6.5 s each, totaling time: 19.5 s. Thereafter, multi-phase dynamic enhanced scanning was performed with the following parameters: flip angle: 10°, number of phases scanned: 35, totaling time: 227.5 s; the remaining parameters were the same as above. Subsequently, a gadolinium contrast agent (Omniscan, GE Healthcare, China) was injected through the median elbow vein using the high-pressure injector in phase 3. The injection dose and speed were 0.1 mmol/kg and 3.5 ml/s, respectively. Finally, 20 ml of saline was injected at the same flow rate for flushing.

### Image Data Analysis and Processing

**FIGURE 1:**
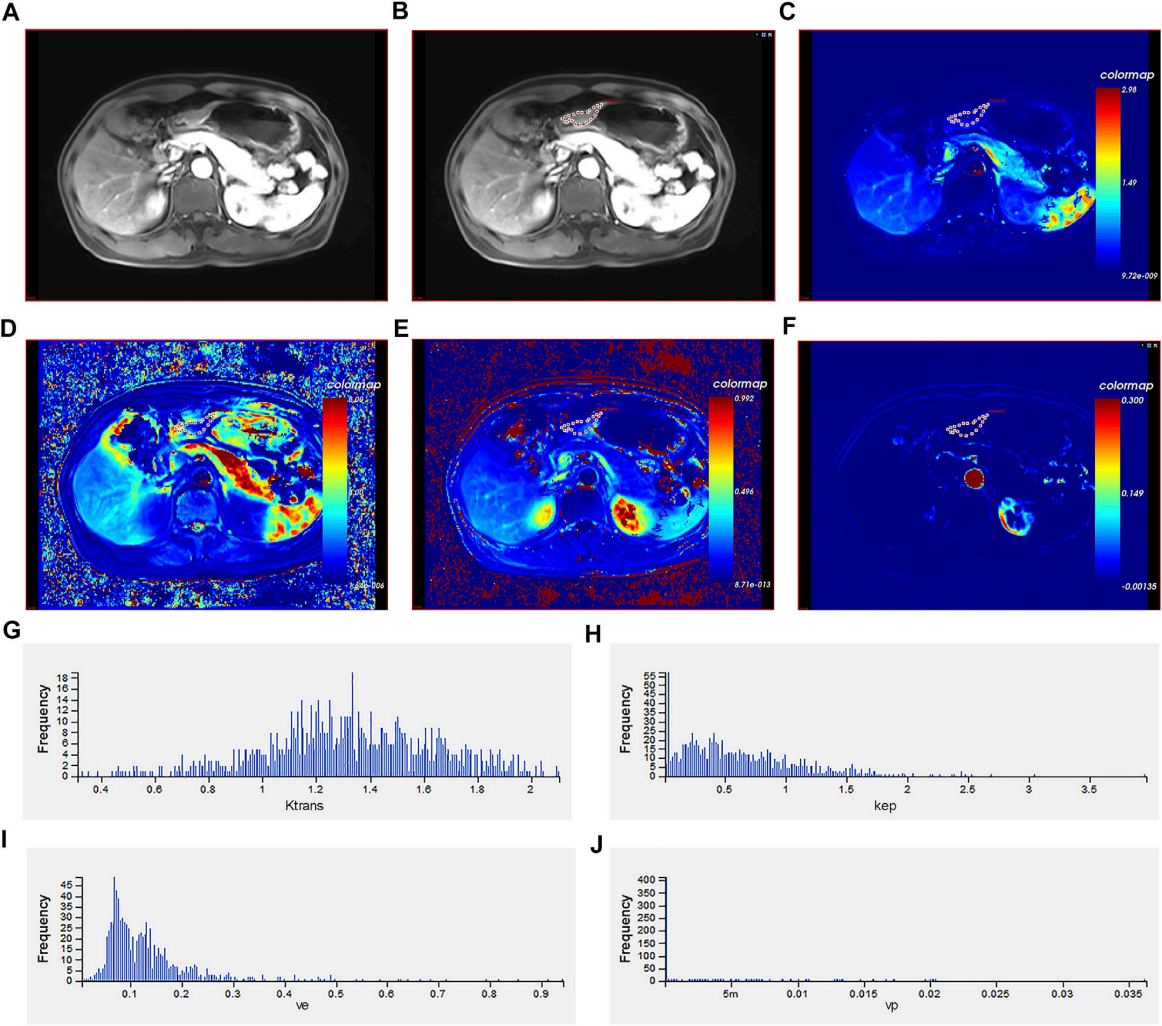
Different imaging modalities and histogram of quantitative perfusion parameters of a 40-year-old male with advanced gastric cancer. **(A)** Axial T1-weighted image showing a mass with irregular and thickened gastric wall. **(B)** Manual ROI placement in the axial T1-weighted image. **(C)** Volume transfer constant (Ktrans) map of ROI. **(D)** Reverse reflux rate constant (Kep) map of ROI. **(E)** Extracellular extravascular volume fraction (Ve) map of ROI. **(F)** Plasma volume fraction (Vp) map of ROI. **(G)** Histogram of Ktrans values. The histogram analysis parameters (min^−1^) were as follows: skewness = 0.980, kurtosis = 0.303, uniformity = 0.451, energy = 0.009, and entropy = 7.060. **(H)** Histogram of Kep values. The histogram analysis parameters (min^−1^) were as follows: skewness = 2.271, kurtosis = 9.384, uniformity = 0.226, energy = 0.120, and entropy = 6.050. **(I)** Histogram of Ve values. The histogram analysis parameters (min^−1^) were as follows: skewness = 2.309, kurtosis = 12.511, uniformity = 0.555, energy = 0.015, and entropy = 6.300. **(J)** Histogram of Vp values. The histogram analysis parameters (min^−1^) were as follows: skewness = 4.985, kurtosis = 24.264, uniformity = −2.596, energy = 0.761, and entropy = 1.334. ROI: region of interest.

The original DCE data was transferred to the Omni Kinetics (GE Healthcare, China) software. First, in order to correct the ROI displacement caused by patients' breathing and other involuntary movements, the DCE images were pre-processed with three-dimensional non-rigid registration. Second, multi-flip angles of 5°, 10°, and 15° and corrected dynamic enhancement sequence scans were processed by the Omni Kinetics software. Further, the arterial input function was performed, and abdominal aorta was selected as the input artery. Third, application of the Tofts model obtained four quantitative parameter maps (Ktrans, Kep, Ve, and Vp) [13]. The volume of interest was designed as a region containing necrotic and cystic tissues to determine the heterogeneity of tumor. Two senior radiologists manually outlined each layer of the AGC lesion in the quantitative parameter maps to form a 3D ROI for calculation. The software then automatically generated several commonly used histogram parameters (mean value; skewness; 10th, 25th, 50th, 75th, and 90th percentiles; uniformity; kurtosis; energy; and entropy) (Figure 1). All calculations were repeated three times to obtain the average value.

### Immunohistochemical Evaluation of Vascular Endothelial Growth Factor and Epidermal Growth Factor Receptor

All GC specimens obtained via surgery or gastroscopy were embedded in paraffin and then cut into 2‐μm‐thick slices. First, antigen retrieval was performed after dewaxing and dehydrating the sections, followed by inhibiting endogenous peroxidase activity using 3% H_2_O_2_ solution at 37 °C for 10 min (H36021594, Nanchang Baiyun Pharmaceutical Co., Ltd., Nanchang, China). Second, the histological slices were stained using the rabbit anti-human VEGF (GT217002, Gene Tech, Shanghai, China) or EGFR (GT209302, Gene Tech, Shanghai, China) and stored in the special refrigerator at 4 °C overnight. Third, all slices were stained with secondary antibody (K5007, Dako, Beijing, China) and then incubated at 37 °C for 30 min. Fourth, DBA staining, rinsing, counterstaining, dehydration, transparency, and mounting were sequentially carried out. Lastly, two senior pathologists independently analyzed all slides and explained the staining results using a microscope, and the staining was explained according to the staining intensity and the percentage of positive cells in the tumor. The scoring method of staining intensity was as follows: zero point: no coloration, one point: light brown, two points: brown, and three points: dark brown. The scoring method of positive tumor cell percentage was as follows: zero points: no positive tumor cell, one point: percentage of positive tumor cells is <10%, two points: percentage of positive tumor cells is 10–50%, three points: percentage of positive tumor cells is 50–80%, and four points: percentage of positive tumor cells is >80%. Finally, the above two scores were multiplied to obtain the immunoreactive score (IRS) [14]. For VEGF, IRS greater or equal to 4 were considered to indicate high expression, whereas scores lower than 4 were considered to indicate low expression. For EGFR, IRS ≥6 and IRS <6 were considered to indicate high expression and low expression, respectively.

### Lauren Classification

Tumor tissue samples were examined by two experienced pathologists who were blinded to patients’ information and samples were classified according to Lauren classification as follows [15]: (1) intestinal-type: preserved tubular or glandular appearance, (2) diffuse-type: no tubular structures and comprised single or small clusters of cells, and (3) mixed-type: combination of diffuse-type and intestinal-type.

### Statistical Analyses

The normality assumption of the above data was assessed using the Kolmogorov–Smirnov test. Continuous variables were presented as mean and standard deviation, and the Mann–Whitney U test and independent sample t-test were used when appropriate. Categorical variables were presented as frequency (%), and the Fisher’s exact test or chi-squared test were used when appropriate. Spearman correlation analysis was used to identify the relationship between VEGF, EGFR, and DCE-MRI perfusion histogram parameters. Statistical analysis and figure creation for the present study was performed using the R software (version 40.2, R Foundation for Statistical Computing, Vienna, Austria), and *p* < 0.05 was considered to be statistically significant.

## Results

### Demographic Characteristics of Patients With Advanced Gastric Cancer

The demographic characteristics of the two groups of patients with different Lauren classifications of AGC are summarized in Table 1. Among the 80 AGC patients, 45 (69.53 years, range 54–88) had pathologically confirmed intestinal-type AGC, and the remaining 35 (65.57 years, range 49–85) had diffuse-type AGC; mixed-type AGC was not observed in the present study. The results indicated that patients with intestinal-type AGC were relatively well-differentiated (*p* < 0.001).

**TABLE 1:** Clinical characteristics of patients with advanced gastric cancers.

Characteristics	Intestinal type (n = 45)	Diffuse type (n = 35)	F value	*p* value
Gender			2.075	0.150
Male	36 (80.0%)	23 (65.7%)
Female	9 (20.0%)	12 (34.3%)
Age (years, x ± s)	69.53 ± 9.40	65.57 ± 11.63	2.040	0.096
Age range	54–88	49–85
Location			0.047	0.997
Cardia	8 (17.8%%)	6 (17.1%)
Body	28 (62.2%)	22 (62.9%)
Antrum	6 (13.3%)	5 (12.3%)
Whole	3 (6.7%)	2 (5.7%)
BMI (Kg/m^2^)	22.41 ± 2.98	21.33 ± 5.89	-0.517	0.607
Differentiation level			28.821	<0.001
High	10 (22.2%)	1 (2.9%)
Moderate	26 (57.8%)	6 (17.1%)
Poor	9 (20.0%)	28 (80.0%)
CEA (ng/ml)			1.512	0.219
Normal	29 (64.4%)	27 (77.1%)
Elevated	16 (35.6%)	8 (22.9%)	
CA199 (µ/ml)			0.369	0.544
Normal	36 (80.0%)	26 (74.3%)
Elevated	9 (20.0%)	9 (25.7%)

### Association Between DCE-MRI Perfusion Histogram Parameters and Various Differentiation Levels

**FIGURE 2:**
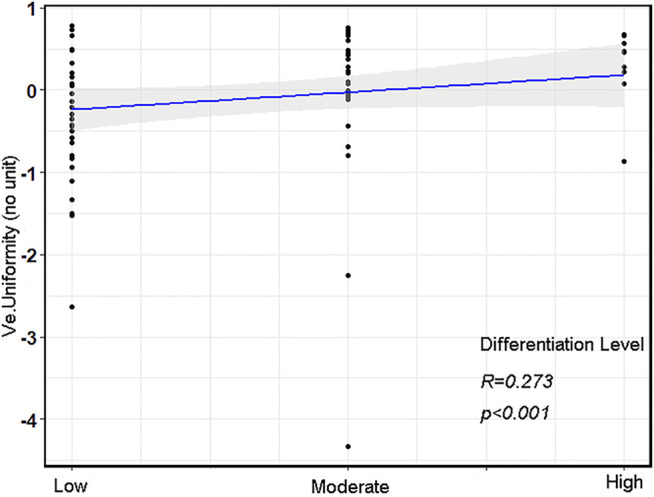
The correlation scatterplot between various differentiation levels and Ve (Uniformity) in advanced gastric cancer (AGC).

Of the aforementioned DCE-MRI perfusion histogram parameters, only Ve (Uniformity) was positively correlated with the differentiation levels (r = 0.273, *p* = 0.014, Figure 2).

### VEGF and EGFR Expressions of AGC

**FIGURE 3:**
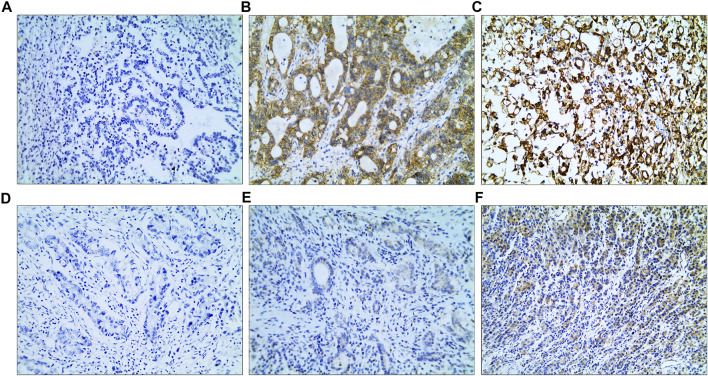
Immunohistochemistry (IHC) images. **(A–C)** IHC images of epidermal growth factor receptor; **(A)** IRS: 0; **(B)** IRS: 8; **(C)** IRS: 12. **(D–F)** IHC images of vascular endothelial growth factor; **(D)** IRS: 0; **(E)** IRS: 2; **(F)** IRS: 8 (magnification: ×10 20). IRS, immunoreactive score.

**FIGURE 4:**
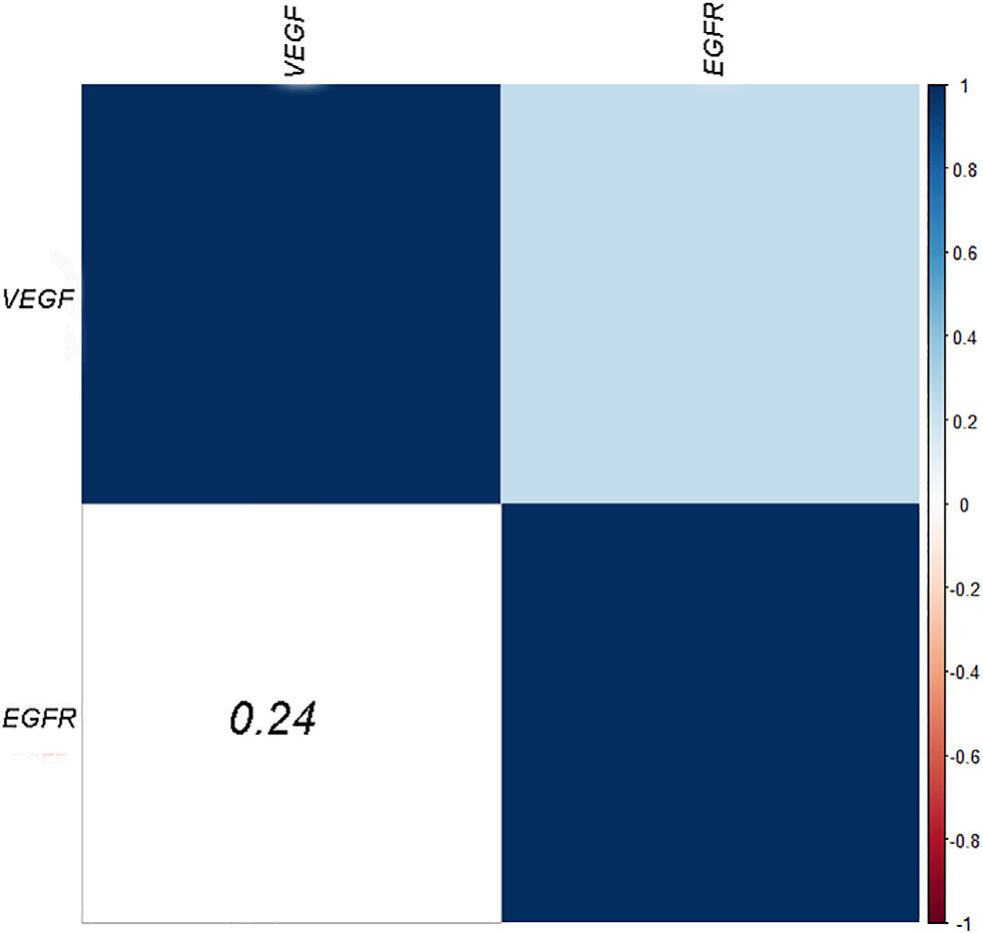
Heatmaps of the correlation between VEGF and EGFR expression.

**FIGURE 5:**
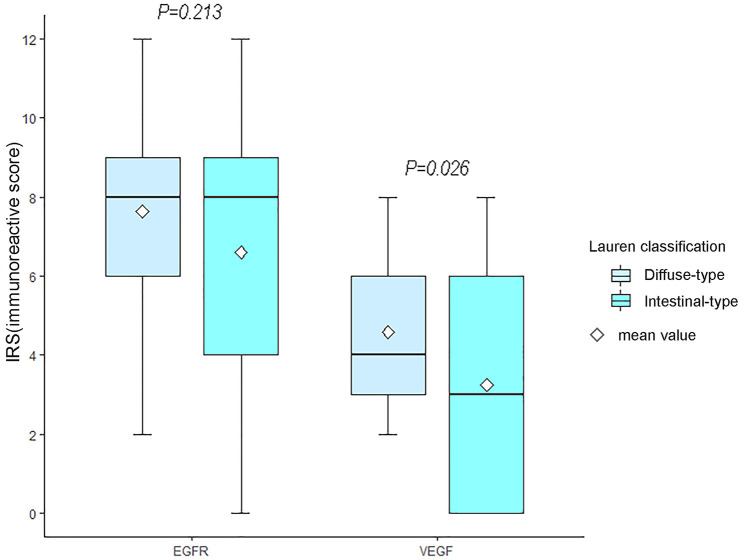
Differences in vascular endothelial growth factor (VEGF) and epidermal growth factor receptor (EGFR) between the two types of advanced gastric cancer (AGC). The expression of VEGF in diffuse-type AGC was higher than that in intestinal-type AGC (*p* = 0.026). Moreover, significant differences in EGFR expression were not observed between intestinal-type and diffuse-type AGC.

VEGF and EGFR expressions were identified by IHC (Figure 3). Correlation analysis revealed a positive correlation between VEGF and EGFR expression, however at a low correlation coefficient value (*p* = 0.033, r = 0.24, Figure 4). As shown in Figure 5, there were significant differences in VEGF expression in the different Lauren classifications of AGC, with the diffuse-type AGC showing a higher expression of VEGF than the intestinal-type. However, the difference in EGFR expression was not statistically significant between the diffuse- and intestinal-type AGC (*p* > 0.05).

### Receiver Operating Characteristics Curve Analysis of DCE-MRI Perfusion Histogram Parameters for Differentiating High Expression Group (VEGF/EGFR) From Low Expression Group in AGC

**FIGURE 6:**
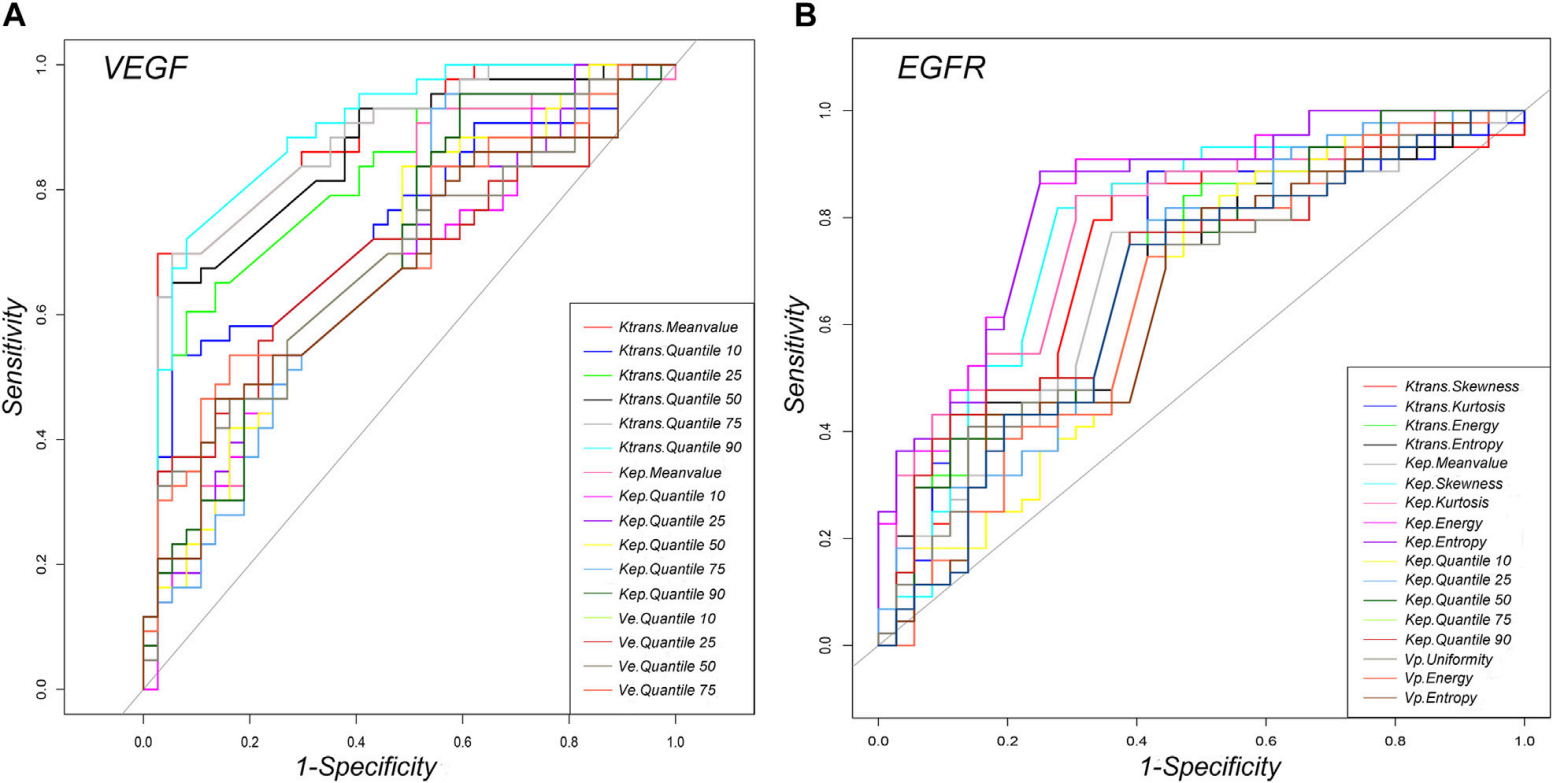
Graphs showing the ROC curves of DCE-MRI perfusion histogram parameters for differentiating high and low **(A)** VEGF and **(B)** EGFR expressions.

For VEGF, ROC curve analysis revealed that Quantile 90 of Ktrans (0.498), Meanvalue of Kep (0.448) and Quantile 50 of Ve (0.696) provided the perfect combination of sensitivity (0.721, 0.907, 0.539), specificity (0.919, 0.486, 0.838), positive predictive value [PPV] (0.912, 0.672, 0.700) and negative predictive value [NPV] (0.739, 0.818, 0.700) for distinguishing high VEGF expression from low expression in AGC (*p* < 0.05 for each item). The area under the curve (AUC) for Ktrans, Kep and Ve were 0.895, 0.695, and 0.700, respectively (Table 2 and Figure 6). In addition, for EGFR, ROC curve analysis revealed that Skewness of Ktrans (0.579), Energy of Kep (0.629) and Entropy of Vp (0.578) provided the perfect combination of sensitivity (0.864, 0.864, 0.750), specificity (0.639, 0.750, 0.611), PPV (0.745, 0.809, 0.702), and NPV (0.793, 0.818, 0.667) for distinguishing VEGF high expression from low expression in AGC (*p* < 0.05 for each item). The AUCs for Ktrans, Kep and Ve were 0.715, 0.836, and 0.660, respectively (Table 3 and Figure 6).

**TABLE 2:** ROC curve analysis of histogram parameters for differentiating VEGF high expression from low expression in AGC.

Kinetic parameter	Histogram metrics	Cut-off	Sensitivity	Specificity	Accuracy	PPV	NPV	AUC	*p* value
Ktrans	Meanvalue	0.549	0.697	0.973	0.825	0.968	0.735	0.880	<0.001
	Quantile 10	0.566	0.535	0.946	0.725	0.920	0.636	0.746	<0.001
	Quantile 25	0.559	0.605	0.919	0.75	0.897	0.667	0.819	<0.001
	Quantile 50	0.593	0.628	0.973	0.788	0.964	0.692	0.859	<0.001
	Quantile 75	0.536	0.698	0.946	0.813	0.938	0.729	0.876	<0.001
	Quantile 90	0.498	0.721	0.919	0.813	0.912	0.739	0.895	<0.001
Kep	Meanvalue	0.448	0.907	0.486	0.713	0.672	0.818	0.695	0.003
	Quantile 10	0.528	0.535	0.730	0.625	0.697	0.574	0.654	0.018
	Quantile 25	0.484	0.837	0.459	0.663	0.643	0.708	0.671	0.009
	Quantile 50	0.470	0.837	0.514	0.688	0.667	0.731	0.688	0.003
	Quantile 75	0.452	0.930	0.459	0.713	0.667	0.85	0.685	0.005
	Quantile 90	0.427	0.953	0.405	0.700	0.651	0.882	0.691	0.003
Ve	Quantile 10	0.489	0.558	0.784	0.663	0.750	0.604	0.692	0.003
	Quantile 25	0.557	0.465	0.838	0.638	0.769	0.574	0.687	0.004
	Quantile 50	0.696	0.539	0.838	0.675	0.793	0.608	0.700	0.003
	Quantile 75	0.540	0.465	0.869	0.650	0.800	0.582	0.673	0.008

**TABLE 3:** ROC curve analysis of histogram parameters for differentiating EGFR high expression from low expression in AGC.

Kinetic parameter	Histogram metrics	Cut-off	Sensitivity	Specificity	Accuracy	PPV	NPV	AUC	*p* value
Ktrans	Skewness	0.579	0.864	0.639	0.763	0.745	0.793	0.715	0.001
	Kurtosis	0.590	0.886	0.583	0.750	0.722	0.808	0.706	0.002
	Energy	0.596	0.795	0.583	0.700	0.700	0.700	0.700	0.002
	Entropy	0.581	0.750	0.583	0.675	0.688	0.656	0.686	0.004
Kep	Meanvalue	0.522	0.773	0.639	0.713	0.723	0.697	0.682	0.005
	Skewness	0.602	0.818	0.722	0.775	0.783	0.767	0.777	<0.001
	Kurtosis	0.624	0.840	0.694	0.775	0.771	0.781	0.778	<0.001
	Energy	0.629	0.864	0.750	0.813	0.809	0.818	0.836	<0.001
	Entropy	0.554	0.886	0.750	0.825	0.813	0.844	0.834	<0.001
	Quantile 10	0.514	0.795	0.528	0.675	0.673	0.679	0.654	0.018
	Quantile 25	0.527	0.773	0.611	0.700	0.708	0.688	0.696	0.003
	Quantile 50	0.526	0.773	0.611	0.700	0.708	0.688	0.694	0.003
	Quantile 75	0.511	0.773	0.611	0.700	0.708	0.688	0.696	0.003
	Quantile 90	0.513	0.750	0.611	0.688	0.702	0.667	0.667	0.001
Vp	Uniformity	0.533	0.795	0.556	0.688	0.686	0.690	0.648	0.023
	Energy	0.507	0.795	0.556	0.688	0.686	0.690	0.650	0.021
	Entropy	0.578	0.750	0.611	0.688	0.702	0.667	0.660	0.014

### Association Between DCE-MRI Perfusion Histogram Parameters and VEGF and EGFR Expressions in Different Lauren Classifications of AGC

**FIGURE 7:**
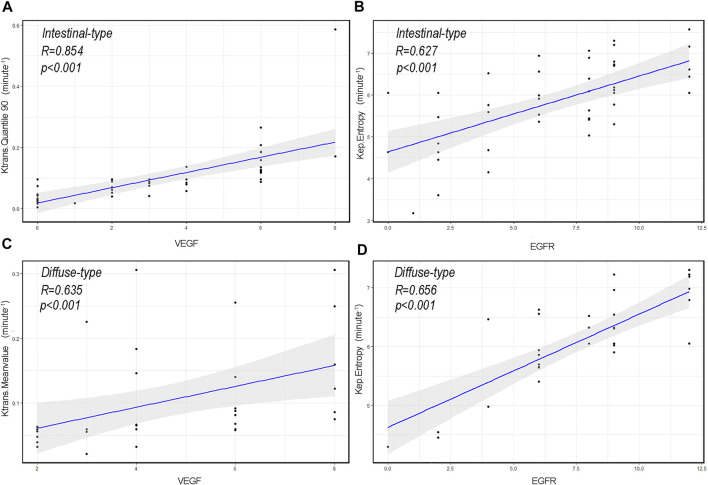
The correlation scatterplot between vascular endothelial growth factor (VEGF), epidermal growth factor receptor (EGFR), and dynamic contrast-enhanced magnetic resonance imaging perfusion histogram parameters of advanced gastric cancer (AGC) in different Lauren classifications. **(A)** Scatter diagram of Quantile 90 of volume transfer constant (Ktrans) in intestinal-type AGC and VEGF expression level (r = 0.854, *p* < 0.001). **(B)** Scatter diagram of entropy of reverse reflux rate constant (Kep) in intestinal-type AGC and EGFR expression level (r = 0.627, *p* < 0.001). **(C)** Scatter diagram of the Meanvalue of Ktrans in diffuse-type AGC and VEGF expression level (r = 0.635, *p* < 0.001). **(D)** Scatter diagram of Entropy of Kep in diffuse-type AGC and EGFR expression level (r = 0.656, *p* < 0.001).

Several perfusion histogram parameters that are significantly correlated to the expression of VEGF and EGFR are summarized in Tables 4 and 5 and Figure 7. In patients with intestinal-type AGC, the correlation coefficient between Quantile 90 of Ktrans and VEGF was the highest (r = 0.854, *p* < 0.001), and Entropy of Kep had the highest correlation coefficient with EGFR (r = 0.627, *p* < 0.001). In patients with diffuse-type AGC, the correlation coefficient between the Meanvalue of Ktrans and VEGF was the highest (r = 0.635, *p* < 0.001), and Entropy of Kep had the highest correlation coefficient with EGFR (r = 0.656, *p* < 0.001).

**TABLE 4:** Association between dynamic contrast-enhanced magnetic resonance imaging perfusion histogram parameters and vascular endothelial growth factor expression in different Lauren classifications of advanced gastric cancer.

Classification	Kinetic parameter	Histogram metrics	R value	*p* value
Intestinal type	Ktrans	Meanvalue	0.829	<0.001
		Quantile 50	0.788	<0.001
		Quantile 75	0.834	<0.001
		Quantile 90	0.854	<0.001
	Kep	Entropy	0.325	0.029
	Ve	Entropy	0.361	0.015
		Quantile 10	0.513	<0.001
		Quantile 25	0.527	<0.001
		Quantile 50	0.513	<0.001
		Quantile 75	0.456	0.002
		Quantile 90	0.349	0.019
	Vp	Skewness	0.469	<0.001
		Kurtosis	0.310	0.038
		Energy	0.522	<0.001
Diffuse type	Ktrans	Meanvalue	0.635	<0.001
		Quantile 25	0.472	0.004
		Quantile 50	0.604	<0.001
		Quantile 75	0.597	<0.001
		Quantile 90	0.599	<0.001
	Kep	Skewness	-0.422	0.012
	Vp	Quantile 10	0.430	0.010
		Quantile 25	0.420	0.012

**TABLE 5:** Association between dynamic contrast-enhanced magnetic resonance imaging perfusion histogram parameters and epidermal growth factor receptor expression in different Lauren classifications of advanced gastric cancer.

Classification	Kinetic parameter	Histogram metrics	R value	*p* value
Intestinal type	Ktrans	Meanvalue	0.315	0.035
		Entropy	0.391	0.008
		Quantile 25	0.326	0.029
		Quantile 50	0.336	0.024
	Kep	Meanvalue	0.392	0.008
		Uniformity	0.351	0.018
		Entropy	0.627	<0.001
		Quantile 50	0.442	0.002
		Quantile 75	0.458	0.002
	Vp	Skewness	0.408	0.005
		Energy	0.379	0.010
Diffuse type	Ktrans	Entropy	0.458	0.006
		Quantile 50	0.341	0.045
	Kep	Meanvalue	0.344	0.043
		Uniformity	0.466	0.005
		Entropy	0.656	<0.001
		Quantile 25	0.415	0.013
		Quantile 50	0.406	0.016

## Discussion

Currently, the commonly used classification systems for AGC outside Japan include the World Health Organization (WHO) classification 2019 and Lauren classification. The WHO classification, which classifies gastric adenocarcinoma into many subtypes, such as tubular, parietal cell, micropapillary, mucinous, poorly cohesive, signet ring cell, hepatoid, mucoepidermoid, medullary carcinoma with lymphoid stroma, and Paneth cell type, has been criticized for its complexity [1]. Thus, the Lauren classification is still the most widely used classification method worldwide since its introduction in 1965 for its high repeatability and simple operability. In the present study, the Lauren classification was chosen, and AGC was divided into intestinal-, diffuse-, and mixed-type [16]. Different histological subtypes of GC proposed by the Lauren classification exhibit distinct clinical and molecular characteristics, such as cell differentiation, biological behaviors, and prognosis [17]. In addition, VEGF and EGFR are important factors that affect the prognosis of AGC. Therefore, clarifying the expression of VEGF and EGFR and their difference in different Lauren classifications may facilitate the identification of high-risk patients and the provision of appropriate treatment.

DCE-MRI, a promising functional imaging technique in oncology, allows for quantitative assessment of functional aspects of tumor microcirculation, such as tumor blood flow, vessel permeability, and vascular and interstitial volumes [18,19]. Previously, DCE-MRI has been proven useful in predicting the histological type and Lauren classification of the tumor and estimating tumor angiogenesis in GC [7]. It can also predict extramural venous invasion preoperatively in local AGC [10]. Unlike the abovementioned studies, which used conventional imaging analysis, we used a histogram analysis based on DCE-MRI in this study. Histogram analysis is a mathematical method that evaluates the distribution of gray-level tones on biomedical images, deriving metrics that reflect the frequency of pixels exhibiting gray levels that lie within a given interval. Therefore, histogram-derived parameters may reflect inner features of tumors that cannot be evaluated through conventional image analysis, such as tumor heterogeneity [20]. Subsequently, the heterogeneity analysis allows for a better understanding of tumor characteristics that may affect treatment planning [21].

This study found that DCE-MRI perfusion histogram parameters had good diagnostic performance in identifying high and low VEGF expressions. ROC curve analysis revealed that Quantile 90 of Ktrans (0.498), Meanvalue of Kep (0.448), and Quantile 50 of Ve (0.696) provided the perfect combination of sensitivity (0.721, 0.907, 0.539), specificity (0.919, 0.486, 0.838), PPV (0.912, 0.672, 0.700), and NPV (0.739, 0.818, 0.700) for distinguishing high and low VEGF expressions in AGC (*p* < 0.05 for each item). The AUC for Ktrans, Kep, and Ve were 0.895, 0.695 and 0.700, respectively. Furthermore, Ktrans (Quantile 90) in intestinal-type AGC and Ktrans (Quantile 50) in diffuse-type AGC had the highest positive correlation with VEGF. Presumably, Ktrans can estimate the combination of blood flow and permeability properties [22]. Compared with normal tissues, tumors require more rigorous supplies of nutrients and oxygen and an enhanced capacity to eliminate metabolic wastes and carbon dioxide. Moreover, VEGF can promote the gene expression of endothelial cells and increase microvascular permeability by stimulating the division and proliferation of vascular endothelial cells [23]. Therefore, the observed relationship between Ktrans (Quantile 90) and Ktrans (Quantile 50) and VEGF expression is reasonable. The findings reported in this study are consistent with those of previous reports in that VEGF and Ktrans have significantly positive associations in gliomas [24,25]. Furthermore, this study indicated that VEGF expression in diffuse-type AGC was significantly higher than that in intestinal-type AGC. One possible explanation for the difference is that, when compared with intestinal-type AGC, diffuse-type AGC is always associated with more aggressive features, including bigger tumor size, poorer tumor differentiation, and increased lymphatic vascular infiltration [26]. Thus, more VEGF is needed to promote angiogenesis. Additionally, Wang et al. [27] indicated that VEGF overexpression is significantly associated with diffuse-type AGC. However, other studies present different views [6,28]. Whether diffuse-type AGC is associated with the overexpression of VEGF requires further investigation due to the relatively small sample size in this study.

EGFR is a type of membrane tyrosine kinase receptor that is overexpressed in 30–60% of GCs, and it initiates an intracellular signal pathway that promotes cancer cell proliferation, cell migration, and angiogenesis [29]. Several experiments have displayed a positive association between EGFR overexpression and drug resistance [30,31]. In this study, ROC curve analysis revealed that Skewness of Ktrans (0.579), Energy of Kep (0.629) and Entropy of Vp (0.578) provided the perfect combination of sensitivity (0.864, 0.864, 0.750), specificity (0.639, 0.750, 0.611), PPV (0.745, 0.809, 0.702), and NPV (0.793, 0.818, 0.667) for distinguishing high and low VEGF expressions in AGC (*p* < 0.05, respectively). The AUC for Ktrans, Kep, and Ve were 0.715, 0.836, and 0.660, respectively. In addition, we found that Kep had the highest positivity associated with EGFR expression in intestinal- and diffuse-type AGC. Kep describes the rate constant of back flux from the extracellular extravascular space (EES) to plasma, which is closely associated with the permeability of the blood vessel wall. Previous studies also indicated that Kep might reflect microvessel density features such as vessel area [32]. Presumably, higher EGFR expression leads to tumors with a more aggressive nature and, thus more angiogenesis, resulting in a positive association between Kep and EGFR. However, the difference was not statistically significant in EGFR (*p* = 0.213) between the two types of AGC, which contradicted our initial expectations. Theoretically, EGFR of the intestinal-type should be relatively lower than that of the diffuse-type because of the more aggressive behavior of the latter. This phenomenon may be interpreted that diffuse-type AGC seems to show low level differentiation and thus the low expression of EGFR. In addition, the correlation analysis in this study demonstrated a positive correlation between VEGF and EGFR expression, suggesting that the interaction between VEGF and EGFR may be involved in the development of AGC. However, the correlation coefficient value is relatively low (r = 0.24). We believe that the study results may be related to the limited number of patients and the heterogeneity in AGC. VEGF expression may be strongly correlated with EGFR expression in some AGC types and weakly correlated in other types. Nevertheless, further prospective and large-scale studies are needed to investigate the relationship between VEGF and EGFR.

According to these results, there was a specific association between the histogram parameters derived from Ve and Vp and VEGF and EGFR expressions. Ve represents the volume of EES per unit volume of tissue, and Vp predominantly reflects the percentage of contrast agents in blood [33]. This study showed that Vp and Ve could also be used as markers for the expressions of VEGF and EGFR in AGC. Furthermore, the results also identified another phenomenon, namely, the difference between intestinal-type and diffuse-type AGC in different tumor grades. Intestinal-type AGC was more well-differentiated than diffuse-type AGC, according to their definitions. Some previous studies have reported the same results [26]. In addition, associations were observed between Ve (Uniformity) and differentiation level in our study (r = 0.273, *p* = 0.014). The differentiation level plays a critical role in tumor prognosis. Patients with high-level differentiation tumors had a lower degree of tumor malignancy and a good prognosis, while lower-level differentiation tumors were associated with a high degree of malignancy and a poor prognosis. And the differentiation level is also associated with the proliferation, invasion, and metastasis of cancer cells [34]. Therefore, identifying the potential biomarkers of tumor differentiation is crucial in determining individual treatment regimens and prognosis. In our study, high-level differentiation AGCs exhibited higher Ve (Uniformity) values than low-level differentiation AGCs, which may be attributed to the more distorted and narrower intercellular spaces of low-level differentiation AGCs. Nevertheless, this association should be further studied for better clarity.

Overall, VEGF and EGFR play essential roles in tumor growth, invasion, metastasis, and angiogenesis. Preoperatively assessing the expression of VEGF and EGFR has an essential influence on the identification of high-risk patients and the choice of treatment strategy. For example, if high-expression (VEGF/EGFR) was indicated for a given patient, appropriate targeted therapies could be applied. This study preliminarily demonstrated the potential of DCE-MRI in diagnosing the expressions of VEGF and EGFR and estimating their difference in different Lauren classifications of AGC. In the future, we plan to include more patients (based on existing studies) and explore the predictive models based on DCE-MRI to predict VEGF and EGFR expression in AGC.

Several limitations of this study should be kept in mind when interpreting these data. First, this was a retrospective study conducted by a single agency. Second, the number of patients enrolled in this study was relatively small. Last, the spatial distribution of perfusion parameters was uneven; thus, it was difficult for samples to accurately match the corresponding ROI. Evidently, prospective and large-scale multi-center studies are required to overcome these limitations and confirm the preliminary results in the future.

## Conclusion

This study showed that a certain correlation exists between the quantitative perfusion histogram parameters derived from DCE-MRI and VEGF and EGFR expressions in AGC. DCE-MRI quantitative perfusion histogram parameters can serve as imaging biomarkers to noninvasively reflect VEGF and EGFR expressions and estimate their difference in different Lauren classifications of AGC, which provides a reference for early diagnosis and individual treatment of AGC patients.

## Data Availability

The datasets presented in this study can be found in online repositories. The names of the repository/repositories and accession number(s) can be found in the article/supplementary material.
